# Glinide, but Not Sulfonylurea, Can Evoke Insulin Exocytosis by Repetitive Stimulation: Imaging Analysis of Insulin Exocytosis by Secretagogue-Induced Repetitive Stimulations

**DOI:** 10.1155/2009/278762

**Published:** 2009-12-28

**Authors:** Kyota Aoyagi, Mica Ohara-Imaizumi, Chiyono Nishiwaki, Yoko Nakamichi, Shinya Nagamatsu

**Affiliations:** Department of Biochemistry, Kyorin University School of Medicine, Mitaka, Tokyo 181-8611, Japan

## Abstract

To investigate the different effects between sulfonylurea (SU) and glinide drugs in insulin secretion, pancreatic *β*-cells were repeatedly stimulated with SU (glimepiride) or glinide (mitiglinide). Total internal reflection fluorescent (TIRF) microscopy revealed that secondary stimulation with glimepiride, but not glucose and mitiglinide, failed to evoke fusions of insulin granules although primary stimulation with glucose, glimepiride, and mitiglinide induced equivalent numbers of exocytotic responses. Glimepiride, but not glucose and mitiglinide, induced abnormally sustained [Ca^2+^]_i_ elevations and reductions of docked insulin granules on the plasma membrane. Our data suggest that the effect of glinide on insulin secretory mechanisms is similar to that of glucose.

## 1. Introduction

Sulfonylurea (SU) and glinide drugs are commonly used for the treatment of type 2 diabetic patients to stimulate insulin release from pancreatic *β*-cells. Both insulinotropic reagents have distinct structures but bind to the same target, SURs, a subunit of the ATP-sensitive K^+^ channel, resulting in the channel closure, which induces depolarization and subsequent Ca^2+^ influx through voltage-gated Ca^2+^ channel, followed by Ca^2+^-dependent exocytosis of insulin granules [1]. 

Despite their similar insulinotropic effects, we found striking differences in the restoration of *β*-cell function in diabetic GK rats [2]. GK rats show abnormal pancreatic islet morphology, and the glucose-induced exocytotic response and the numbers of docked insulin granules on the plasma membrane are impaired in pancreatic *β*-cells cultured from GK rats. We found that long-term treatment with nateglinide, a glinide drug, caused partial recovery of these impairments in GK rats; the glucose-induced exocytotic response in *β*-cells cultured from GK rats treated with nateglinide for 6 weeks was partially recovered. In addition, the glinide-treatment almost restored the pancreatic islet morphology and the number of docked insulin granules on the plasma membrane. On the other hand, long-term treatment with glibenclamide, an SU drug, did not restore the *β*-cell functions in GK rats. 

One of the explanations for their different effects on the recovery of *β*-cell function would be the different potency of SU and glinides. While SUs bind tightly to SURs and exhibit a delayed onset and prolonged hypoglycemic effects, glinides have a rapid onset and short acting effects on insulin secretion [3]. Because several reports showed that chronic SU treatment induced unresponsiveness of *β*-cells to SU drugs in vivo and in vitro [4, 5], it has been suggested that SU stimulation could cause an impairment of insulin-secretory mechanism in *β*-cells. 

To examine the effects of these drugs on the exocytotic response in *β*-cells, we studied the reversibility of insulin-secreting responses to repetitive glucose, SU, or glinide stimulations in primary cultured mouse pancreatic *β*-cells. 

## 2. Materials and Methods

### 2.1. Total Internal Reflection Fluorescent (TIRF) Microscopy Analysis

Pancreatic *β*-cells were cultured from C57BL/6 mice, infected with recombinant adenovirus to express GFP-tagged insulin and observed under the Olympus TIRF microscope with a high-aperture objective lens as described previously [2]. Cultured *β*-cells were incubated for 15 minutes at 37°C in Krebs-Ringer buffer (KRB) containing (in mM): 110 NaCl, 4.4 KCl, 1.45 KH_2_PO_4_, 1.2 MgSO_4_, 2.3 calcium gluconate, 4.8 NaHCO_3_, 4 glucose, 10 HEPES, pH 7.4, and 0.3% bovine serum albumin (BSA). Cells were then perifused with KRB with 4 mM glucose at a flow rate of 0.5 mL/min and stimulated with KRB containing 16.7 mM glucose, 5.5 mM glucose supplemented with 0.5 *μ*M mitiglinide or glimepiride for 12.5 minutes. After the first stimulation, KRB buffer containing 4 mM glucose was perifused for 15 minutes, followed by the second stimulation with 16.7 mM glucose, 5.5 mM glucose supplemented with 0.5 *μ*M mitiglinide or glimepiride for 12.5 minutes. The numbers of fusion events during the first and second stimuli and the numbers of docked insulin granules just before the first and second stimulations were manually counted.

### 2.2. Measurement of [Ca^2+^]_i_

Fura-2 acetoxymethyl ester (Fura-2 AM; Invitrogen) was loaded into cultured *β*-cells and the ARGUS/HiSCA system (Hamamatsu photonics) was used for [Ca^2+^]_i_ measurement as previously described [2]. Cells were repeatedly stimulated as described above.

### 2.3. Statistical Analyses

Data were analysed by one-way ANOVA followed by Turky-Kramer's test using the Statview software (SAS Institute, Cary, NC, U.S.A.).

## 3. Results

**Figure 1:**
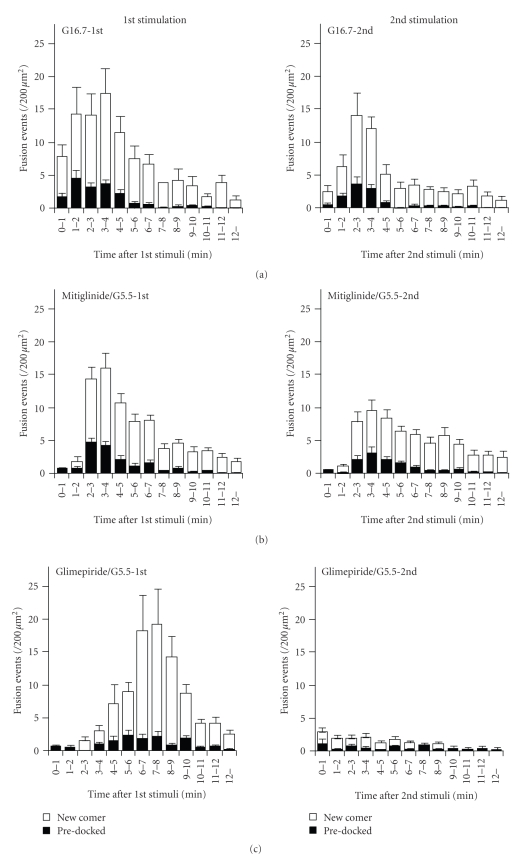

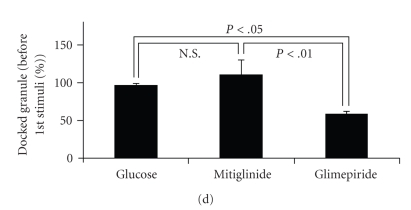
Histogram of the numbers of fusion events (/200 *μ*m^2^) at 1-minute intervals after stimulation. Cells were perifused with 16.7 mM glucose (*N* = 16) (a), 0.5 *μ*M mitiglinide (*N* = 17) (b), and 0.5 *μ*M glimepiride (*N* = 11) (c) for 12.5 minutes. After the first stimulation, cells were perifused with 4 mM glucose for 15 minutes and stimulated again by the same secretagogues as in the first stimulation. Left and right columns show exocytotic responses during thefirst and second stimulations, respectively. (d) The numbers of total fusion events during the first and second stimuli. Data are represented as mean ± S.E.M per 200 *μ*m^2^.

First, we performed repetitive stimulations using physiological stimulation, high glucose. Primary cultured mouse pancreatic *β*-cells expressing GFP-tagged insulin were stimulated twice by perifusion with 16.7 mM glucose, and the exocytotic responses to the first and second stimulations were observed under TIRF microscopy. Figure 1(a) shows the numbers of exocytotic events from pre-docked and newcomer granules in response to the first and second high glucose stimuli. The patterns of exocytotic events during the first and second stimulations were similar indicating that 16.7 mM glucose could repeatedly evoke qualitatively similar exocytotic responses. 

Next, we examined whether the repetitive exocytotic response could be induced by commonly used insulinotropic reagents, SU, and glinide drugs. Glimepiride and mitiglinide were used as representative SU and glinide drugs, respectively. As shown in Figure 1(b), both the first and second mitiglinide stimulations quickly elicited exocytotic responses after the onset of stimulation, although the number of total fusion events evoked by the second mitiglinide stimulus was mildly reduced. On the other hand, glimepiride, surprisingly, failed to evoke the exocytotic response during second stimulus (Figure 1(c)). The number of total fusion events induced by the first glimepiride stimulus was equivalent to those induced by the first 16.7 mM glucose or mitiglinide stimulations (Figure 1(d), 83.0 ± 7.9, 77.7 ± 6.4 and 91.3 ± 13.3/200 *μ*m^2^ for 16.7 mM glucose, mitiglinide and glimepiride, resp.) indicating that the first glimepiride stimulation affected the ability of the response to the subsequent glimepiride stimulus even after a 15-minute restoration period, whereas 16.7 mM glucose and mitiglinide could repeatedly evoke the exocytosis of insulin granules. In order to rule out the possibility that the excess of glimepiride caused the failure of the exocytotic response to the second stimulation, we examined the effect of a lower concentration on repetitive stimulation. Primary stimulation with 0.1 *μ*M glimepiride induced about half the number of fusion events in 12.5 minutes (48.7 ± 6.2/200 *μ*m^2^), but secondary stimulation with 0.1 *μ*M glimepiride failed to evoke a significant exocytotic response (14.9 ± 2.3/200 *μ*m^2^). 

**Figure 2:**
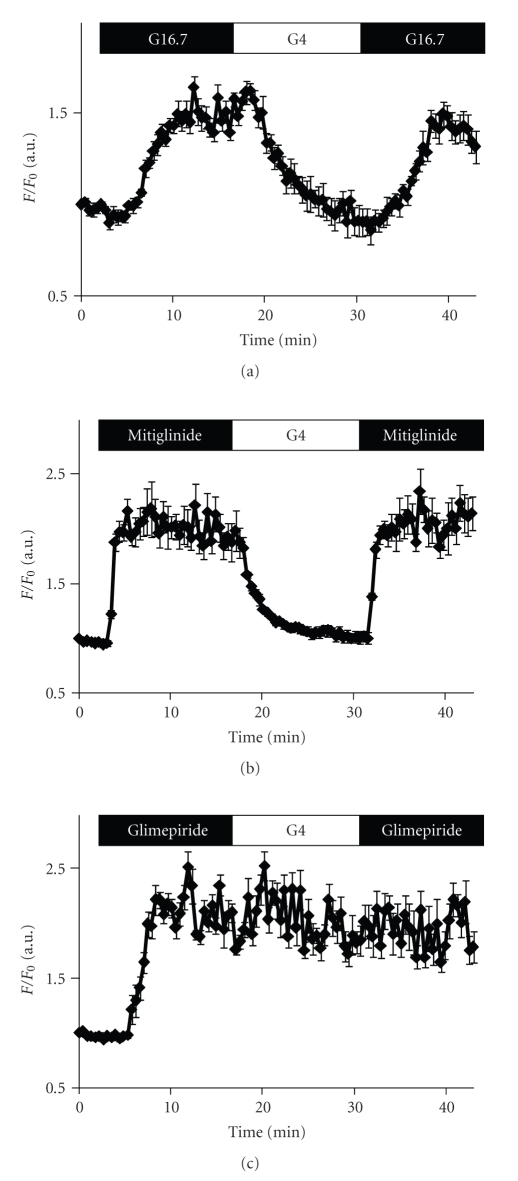
[Ca^2+^]_i_ responses to repetitive stimulations. Cells were perifused with 4 mM glucose and then stimulated with 16.7 mM glucose (a), 0.5 *μ*M mitiglinide (b), or 0.5 *μ*M glimepiride (c) for 12.5 minutes. After the first stimulation, cells were perifused with 4 mM glucose for 15 minutes and stimulated again by the same secretagogues as in the first stimulation. Data are represented as a ratio of the fluorescence intensity expressed as *F*/*F*_0_, where *F*_0_ is the ratio of 340 to 380 nm at the baseline. (mean + S.E.M., *N* = 9, 7, and 7 for 16.7 mM glucose, mitiglinide, and glimepiride, resp.).

To elucidate the mechanism underlying the glimepiride-induced unresponsiveness to the second stimulation, we examined the [Ca^2+^]_i_ dynamics during repetitive stimulations. As shown in Figure 2, the increased [Ca^2+^]_i_ elicited by the first 16.7 mM glucose returned to the basal level during a 15-minute interval, and the second high glucose stimulation again evoked an increase in [Ca^2+^]_i_. Mitiglinide induced a more rapid response than high glucose. The increased [Ca^2+^]_i_ steeply decreased to the basal level and the second mitiglinide stimulation quickly evoked a [Ca^2+^]_i_ increase. On the other hand, the elevated [Ca^2+^]_i_ induced by the first glimepiride stimulation did not return to the basal level during a 15-minute interval, and the second glimepiride stimulation failed to cause a further increase in [Ca^2+^]_i_. Continuous washing for more than 30 minutes also failed to reduce the [Ca^2+^]_i_ evoked by the first glimepiride stimulation (data not shown). These results suggest that the first glimepiride stimulation elicited a high [Ca^2+^]_i_ state for an abnormally long time even after the withdrawal of glimepiride, resulting in the failure of insulin release in response to subsequent stimulations. 

**Figure 3:**
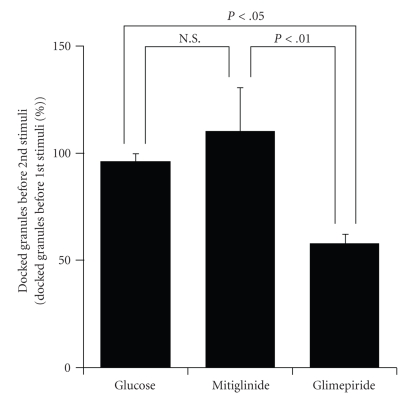
The numbers of docked insulin granules just before the second stimulation. Results are mean ± S.E.M percentage of the numbers of docked insulin granules just before the first stimulation (*N* = 12, 9, and 10 for 16.7 mM glucose, mitiglinide, and glimepiride, resp.).

It is of note that the second glimepiride stimulation failed to induce the exocytotic response even though [Ca^2+^]_i_ during the second stimulus was equivalent to that induced by the first glimepiride stimulation. Thus, we assumed that the abnormally sustained [Ca^2+^]_i_ elevation induced by glimepiride would affect the exocytotic process that is probably involved in the regulation of insulin granule motility, because the exocytotic responses evoked by glimepiride were largely composed of newcomer granules which must move a long distance from the cytosol to the plasma membrane. To this end, we investigated the numbers of docked insulin granules on the plasma membrane after a 15-minute interval following the first stimulation because the motility of insulin granules should be reflected by the number of docked insulin granules after the onset of stimulation [6]. As shown in Figure 3, the first high glucose and mitiglinide stimuli did not affect the numbers of docked insulin granules on the plasma membrane. On the other hand, the number of docked insulin granules was decreased to 58.0 ± 4.5% by the first glimepiride stimulation despite the 15-minute recovery period, suggesting that glimepiride, but not mitiglinide, would impair the intracellular motility of insulin granules and their recruitment to the plasma membrane. These results suggest that the abnormally sustained [Ca^2+^]_i_ elevation by primary glimepiride stimulation impaired insulin granule motility, which might be the cause of the unresponsiveness to the second glimepiride stimulation.

## 4. Discussion

In the present study, we examined the effects of SU and glinide drugs on the reversibility of insulin-secreting response and showed that primary stimulation with SU impaired the exocytotic response to subsequent stimulation, while glucose and glinide had the ability to repeatedly induce insulin release. 

Previous pharmacological studies showed that the IC50 values of glimepiride and mitiglinide to exogenously expressed Kir6.2 and SUR1 were 3.0 nM and about 60 nM, respectively [7]. However, in this study, we used mitiglinide and glimepiride at the same concentration (0.5 *μ*M) because of the following two reasons. First, our previous study showed that the maximal insulinotropic effect of mitiglinide was observed at >0.5 *μ*M under TIRF microscopy, indicating that 0.5 *μ*M mitiglinide induced a moderate exocytotic response in primary cultured *β*-cells [8]. Second, the number of total fusion events evoked by the first 0.5 *μ*M glimepiride stimulation was comparable to that evoked by the first 0.5 *μ*M mitiglinide stimulation (Figure 1(d)) suggesting that the maximum insulin-secreting response should not be induced by 0.5 *μ*M glimepiride. Therefore, in our experimental condition, the insulinotropic potencies of 0.5 *μ*M mitiglinide and 0.5 *μ*M glimepiride were equivalent. The validity of the dosages of mitiglinide and glimepiride used in this study was also proven by the pattern of fusion events in response to the first stimulation. The first glimepiride stimulation evoked fusion events with the highest peak at 7-8 minutes after the onset of stimulation, whereas the first mitiglinide stimulation induced a more rapid response. These results were consistent with previous studies showing that glinide drugs induce a more rapid response than SU [3]. Furthermore, we confirmed that the first stimulation with glimepiride at a lower concentration induced about half the number of total fusion events but impaired the insulin-secreting response to the second stimulation. Taken together, the different exocytotic responses evoked by glimepiride and mitiglinide were attributed not to the difference in the effective concentration but in the character of these drugs. 

It is important to elucidate the mechanism underlying the glimepiride-induced unresponsiveness to subsequent stimulation. We and others showed that glimepiride induced an elevation of [Ca^2+^]_i_ that did not return to the basal level even after the withdrawal of glimepiride (Figure 2(c)) suggesting that an abnormally sustained [Ca^2+^]_i_ elevation might be the cause of the unresponsiveness to subsequent stimulation. It was reported that the abnormally sustained [Ca^2+^]_i_ elevation induced by Ca^2+^ ionophore attenuates the mobility of secretory granules via disruption of the cytoskeleton in astrocyte [9]. Several studies have demonstrated that [Ca^2+^]_i_ elevation activates multiple pathways for the disruption of the cytoskeleton, which may lead to attenuation of the insulin granule mobility. Calcium activates Ca^2+^-dependent protease, calpain, which cleaves microtubule-associated proteins (MAPs), resulting in destabilization of microtubule filaments [10]. In addition, an increase in [Ca^2+^]_i_ stimulates Ca^2+^-dependent phosphatase, calcineurin, followed by cofilin dephosphorylation via slingshot-mediated pathway, which leads to depolymerization of actin filaments [11]. Thus, we suppose that primary stimulation with glimepiride might attenuate the insulin granule mobility via disruption of the cytoskeleton. 

In the present study, dispersed primary cultured *β*-cells were repeatedly stimulated by perifusion with 16.7 mM glucose. Although the proportion of fusion events from pre-docked granules was mildly reduced compared to our previous studies [6], this was due to the different stimulation protocols with a relatively milder stimulation than in our previous studies [2, 6, 8]. Because fusions from pre-docked granules require rapid and marked elevation in [Ca^2+^]_PM_ [12], perifusion with 16.7 mM glucose might not evoke the steep elevation of [Ca^2+^]_PM_ required for fusions from pre-docked granules. 

Several decades ago, it was reported that a first pulse of glucose enhanced insulin secretion in response to a second stimulation. The priming effect of glucose is known as “time-dependent potentiation (TDP)” and is observed in isolated pancreas [13, 14] and islets [15, 16]. In this study, the first and second 16.7 mM glucose induced qualitatively the same exocytotic pattern, but the total number of fusion events induced by the second 16.7 mM glucose was slightly decreased rather than potentiated. This discrepancy might be due to the different preparation for insulin release assay because, to our knowledge, there is no report showing TDP in dispersed *β*-cells. In this study, we used dispersed pancreatic *β*-cells cultured for 2 days in vitro. Thus, cell-cell interaction or communication in pancreatic islets might be important for the establishment of TDP. In line with this speculation, some reports showed the importance of factors derived from non-*β*-cells in the formation of “glucose-memory” [16, 17]. 

In conclusion, mitiglinide is more beneficial than SU not only in improving insulin exocytosis but also in the precise management of [Ca^2+^]_i_, which would be important for maintenance of the insulin-secreting mechanisms in *β*-cells.
